# A Retrospective Description of Pediatric Hepatitis C in a Romanian Cohort: Liver Fibrosis at Diagnosis

**DOI:** 10.3390/diagnostics16060927

**Published:** 2026-03-20

**Authors:** Daniela Păcurar, Alexandru Dinulescu, Irina Dijmărescu

**Affiliations:** 1 Faculty of Medicine, “Carol Davila” University of Medicine and Pharmacy, 050474 Bucharest, Romania; daniela.pacurar@umfcd.ro (D.P.); irina.dijmarescu@umfcd.ro (I.D.); 2 Department of Pediatrics, Emergency Hospital for Children “Grigore Alexandrescu”, 011743 Bucharest, Romania

**Keywords:** pediatric hepatitis C virus (HCV) infection, chronic hepatitis C, children hepatitis

## Abstract

**Background:** Pediatric hepatitis C virus (HCV) infection is often asymptomatic but may lead to significant liver disease later in life. In Romania, data on pediatric HCV remains scarce. This study aimed to describe the clinical and epidemiological characteristics of children with chronic HCV infection in a Romanian cohort. **Methods**: We conducted a retrospective study that included 83 pediatric patients evaluated for chronic hepatitis C between 1995 and 2024 at a tertiary pediatric hospital from Bucharest, Romania. Demographic data, routes of transmission, biochemical parameters, viral load, and liver fibrosis assessed by FibroScan^®^ or liver biopsy were analyzed. **Results**: The median age at diagnosis was 73 months (IQR 36–156), with a slight female predominance (54.2%). Vertical transmission was the most common (48.2%). Most children had normal or mildly elevated transaminases at diagnosis. Although pediatric HCV hepatic involvement is generally considered mild, in our cohort only 40.6% of children had absent or mild fibrosis at diagnosis, while in 33.7% of cases moderate fibrosis was identified, and 8.4% had severe fibrosis or cirrhosis. No significant correlations were found between viral load, transaminase levels, and fibrosis severity. **Conclusions**: Pediatric HCV infection in Romania is frequently diagnosed late, mainly due to the lack of systematic perinatal screening. Although liver disease is generally mild, the cases of advanced fibrosis highlight the need for early diagnosis and improved screening strategies.

## 1. Introduction

Hepatitis C virus (HCV) infection remains a major global health issue. By the latest estimation of the World Health Organization (WHO), there are 50 million people affected worldwide, including approximately 3.2 million children and adolescents [1,2]. The WHO has committed to eradicating viral hepatitis by the year 2030 [3].

In the pediatric population, chronic HCV infection is often silent for years, yet later in life it can lead to progressive liver damage, including fibrosis, cirrhosis, and, albeit rarely, hepatocellular carcinoma [4,5]. Chronic hepatitis C is diagnosed if HCV ribonucleic acid (RNA) is positive for more than 6 months. The latest studies report that approximately one third of the HCV-positive pediatric patients develop severe liver damage in the third decade of life [5,6,7]. The transmission of HCV can be vertical, from mother to child (perinatal transmission), or horizontal, through unsafe medical procedures, intravenous drug use or sexual transmission [8,9,10].

Current international guidelines, including those from the European Association for the Study of the Liver (EASL) and the American Association for the Study of Liver Diseases (AASLD), recommend treatment for all children aged 3 years and older when diagnosed with chronic HCV infection, using approved interferon-free direct-acting antiviral (DAA) regimens, which have shown sustained virologic response (SVR) rates exceeding 95% and excellent safety profiles [11,12]. However, real-world data from Eastern Europe regarding access to and outcomes of these therapies in children remain limited.

Previously, Romania had one of the highest chronic HCV infection prevalence rates in Europe, which is currently significantly decreasing. The prevalence was estimated at 5.9% in 1990 and at 3.2% in 2010, while the latest data from 2023 reports a prevalence of 0.9%, still much higher than the average reported in developed countries (0.05–0.36%) [13,14,15,16,17]. Despite this, data regarding pediatric HCV infections remain scarce, and national screening programs targeting at-risk children are lacking. The absence of routine perinatal HCV screening and limited access to specialized care may contribute to underdiagnosis and delayed treatment in this vulnerable group.

This retrospective study aims to describe the clinical and epidemiological features of pediatric hepatitis C in a Romanian cohort, with focus on potential transmission routes and diagnostic timelines. By providing insight into the local context, we hope to inform future public health strategies to improve early identification and management of HCV in children.

## 2. Materials and Methods

### 2.1. Study Design and Setting

This retrospective cohort study included 83 pediatric patients diagnosed with chronic hepatitis C between 1995 and 2024. All children were evaluated at “Grigore Alexandrescu” Emergency Hospital for Children in Bucharest, Romania, a tertiary referral center for pediatric hepatology, supporting the capital city and surrounding regions.

### 2.2. Study Population and Cohort Development

Patients were identified using electronic databases and hospital medical records. Children were referred for evaluation due to perinatal exposure to HCV, abnormal liver function tests, or incidental detection of anti-HCV antibodies during routine medical assessments. Chronic HCV infection was defined as detectable HCV RNA persisting for more than six months. Only patients with confirmed chronic HCV infection and available clinical data at the time of diagnosis were included in the analysis.

### 2.3. Case Identification and Diagnostic Testing

**Figure 1:**
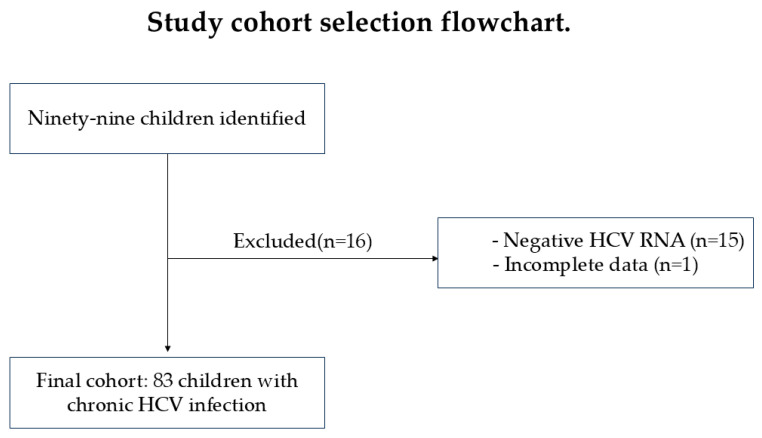
Cohort selection.

Two categories of patients were monitored: patients older than 18 months who tested positive for anti-HCV antibodies following screening tests and exposed patients aged 2–18 months, whose mothers were found to be anti-HCV antibody-positive during pregnancy or at delivery. For each patient category, specific confirmatory tests were performed to either confirm or rule out infection. In exposed patients aged 2–18 months, the viral load was directly assessed through Nucleic Acid Amplification Testing (NAT), without the need for anti-HCV antibody testing. Among the 99 initially identified children, 16 were excluded due to negative HCV RNA results or incomplete follow-up data, resulting in a final cohort of 83 children with confirmed chronic HCV infection (Figure 1).

### 2.4. Serological and Molecular Diagnostic Methods

Anti-HCV antibody testing was performed using commercially available third-generation and later fourth-generation enzyme-linked immunosorbent assays (ELISA), according to the diagnostic standards in use at the time of evaluation. Over the extended study period (1995–2024), assay platforms evolved in line with technological advancements and national laboratory regulations. Reactive anti-HCV results were confirmed according to contemporaneous laboratory protocols and were interpreted solely as markers of exposure to HCV infection.

Detection and quantification of HCV RNA were performed using reverse-transcription polymerase chain reaction (RT-PCR)-based molecular assays validated for clinical use. Throughout the study period, assay sensitivity improved in accordance with updated laboratory standards. Viral load results were expressed in international units per milliliter (IU/mL). Chronic HCV infection was defined as detectable HCV RNA persisting for more than six months, in line with international diagnostic criteria.

### 2.5. Data Collection

We collected demographical data (age, sex, year of diagnosis, area of provenance), epidemiological data (route of transmission, co-infections) and laboratory data (transaminases level, viral load), as well as the results of tests applied for fibrosis evaluation at the time of diagnosis. The cut-off value for detectable viremia in our laboratory was 15 IU/mL.

### 2.6. Liver Fibrosis Assessment Methods

Liver fibrosis was assessed using transient elastography (FibroScan^®^, Echosens, Paris, France) or liver biopsy when clinically indicated. Liver stiffness measurements (LSM) obtained by FibroScan^®^ were expressed in kilopascals (kPa). The following cut-off values were applied for fibrosis staging:

F0–F1 (no to mild fibrosis): <7.1 kPa;

F2 (moderate/significant fibrosis): 7.1–9.4 kPa;

F3 (severe/advanced fibrosis): 9.5–12.4 kPa;

F4 (cirrhosis): ≥12.5 kPa.

These thresholds were selected based on published pediatric studies evaluating transient elastography in chronic viral hepatitis and were aligned with the technical specifications and interpretation standards used at our institution during the study period. Although pediatric-specific liver stiffness cut-offs are not yet fully standardized, the selected ranges fall within the values commonly reported in children with chronic HCV infection [18,19].

### 2.7. Statistical Analysis

The data was analyzed using IBM SPSS Statistics version 25 and illustrated using Microsoft Office Excel/Word 2013. Quantitative variables were tested for normal distribution using the Shapiro–Wilks test and were reported as medians with interquartile ranges (IQR). Quantitative variables were tested between independent groups using Mann–Whitney U tests. The Kruskal–Wallis test was used to determine significant differences between two or more groups of an independent variable. Fisher’s exact test was used to determine the nonrandom associations between categorical variables with Bonferroni method used for correction, and the Bonferroni correction was applied by dividing the conventional significance threshold (α = 0.05) by the number of comparisons, yielding adjusted *p*-value thresholds reported for each set of tests. The Spearman correlation coefficient was used to identify a correlation between 2 non-normal distributed continuous variables (R).

## 3. Results

### 3.1. Patient Demographics

**Figure 2:**
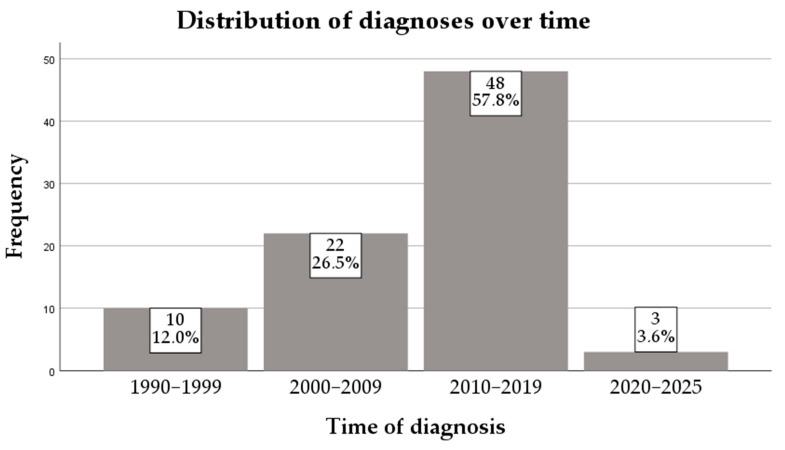
Distribution of diagnoses over time.

This study has included 83 patients. Most patients were from 2010–2019 (57.8%), followed by those from 2000–2009 (26.5%). The distribution of cases over the years is presented in Figure 2.

The majority (74.7%) of patients were from urban areas. There was a slight female predominance (54.2%). Median age at diagnosis was 73 months (36–156). There were 14 children (16.9%) under 2 years old and 69 (83.1%) over 2 years old (Table 1).

### 3.2. Transaminase Levels

**Figure 3:**
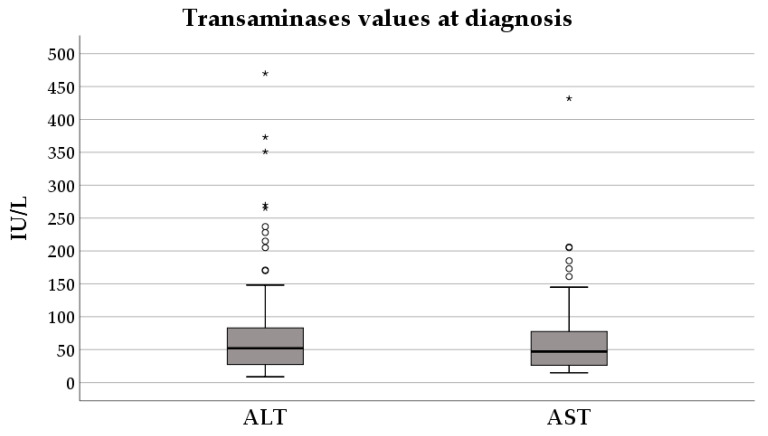
Transaminases values at diagnosis. The interior bars indicate the medians, while the whiskers extend to the maximum and minimum of the data; ◦ = outlier; * = extreme outlier.

The median value of alanine aminotransferase (ALT) at diagnosis was 52 (27–84) IU/L, and for aspartate transaminase (AST) it was 47 (26–79) IU/L (Figure 3).

Most of the children included in the study group had transaminase values within the normal range—48.2% had normal ALT. Mild elevations, defined as an increase of 1–1.5 times the upper limit of normal (ULN), were noted in 14.5% of cases for ALT and 19.3% of cases for AST. Moderate increases, defined as 1.5–3 times the ULN, occurred in 21.7% of patients for ALT and 13.3% for AST, while more important elevations, defined as an increase of 3–5 times the ULN, were less frequent, observed in 8.4% for ALT and 4.8% for AST, respectively. Severe elevations were rare, with ALT and AST increases of 5–10 times the ULN in 6.0% of cases and 1.2% of cases, respectively. Levels more than 10 times the ULN were found only in 1.2% of children.

### 3.3. Viral Load

**Figure 4:**
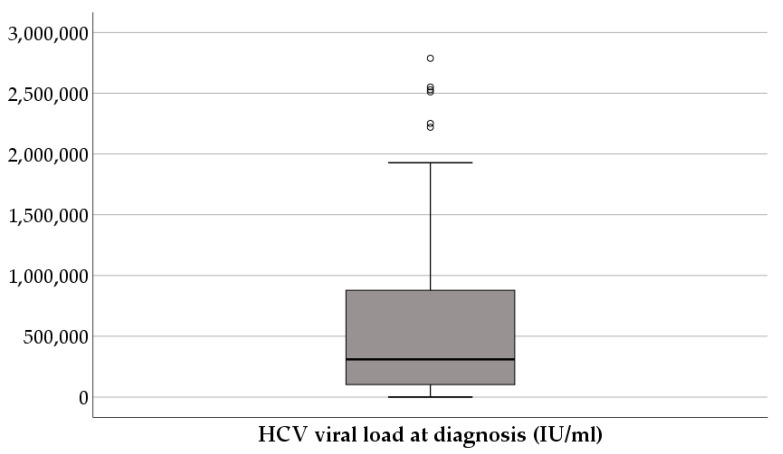
HCV viral load at diagnosis.

The viral load at diagnosis had a median of 3 × 10^5^ (1.1 × 10^5^–9 × 10^5^) IU/mL (Figure 4).

**Figure 5:**
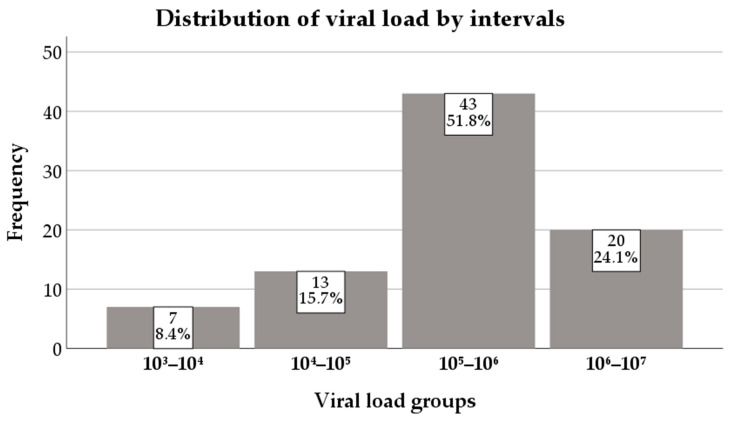
Distribution of viral load by intervals.

Most of the patients had viremia values between 10^5^ and 10^6^ (51.8%), followed by those in the 10^6^–10^7^ group (24.1%) (Figure 5).

### 3.4. Liver Fibrosis Assessment

Fibrosis was evaluated in 69 (83.1%) children—for 10 (14.5%) through liver biopsy and for 59 through FibroScan^®^ (85.5%). Most of them, 34 (40.6%), had insignificant or mild liver scarring, 28 (33.7%) had moderate liver scarring, 6 (7.2%) presented severe liver scarring, and 1 (1.2%) had cirrhosis. The one that had cirrhosis at the time of diagnosis was an adolescent boy with unknown route of transmission. All the patients for which fibrosis was evaluated based on liver biopsy had insignificant or mild fibrosis.

### 3.5. Route of Transmission

**Figure 6:**
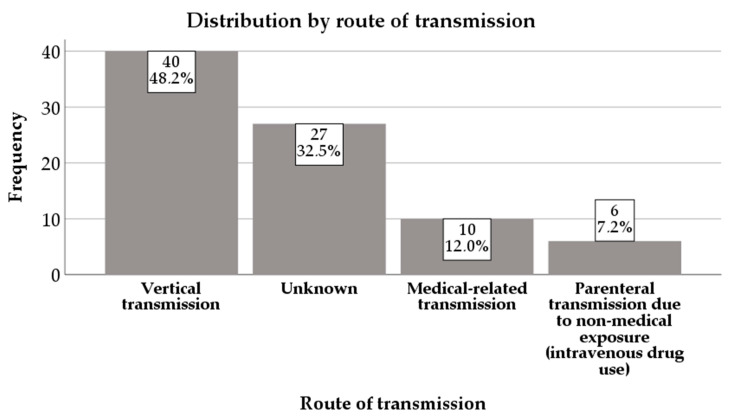
Distribution by route of transmission.

Most patients—40 (48.2%)—had a vertical route of transmission, while for 27 (32.5%) children, the route of transmission was unknown. Ten (12%) children had medical-related transmission and six (7.2%) were infected through intravenous drug use (Figure 6).

Four (4.2%) patients included in the study group had HBV coinfection, all being healthy carriers.

The descriptive statistics of the study subjects is presented in Table 2.

There was no statistically significant association detected between the values of transaminases and age, sex, area of provenance, fibrosis severity, or route of transmission (*p* > 0.05). Additionally, we found no correlation between transaminases and viremia values at diagnosis (*p* > 0.05).

There was no statistically significant association detected between HBV coinfection and age, transaminases and viremia values at diagnosis, fibrosis severity, or route of transmission (*p* > 0.05).

**Figure 7:**
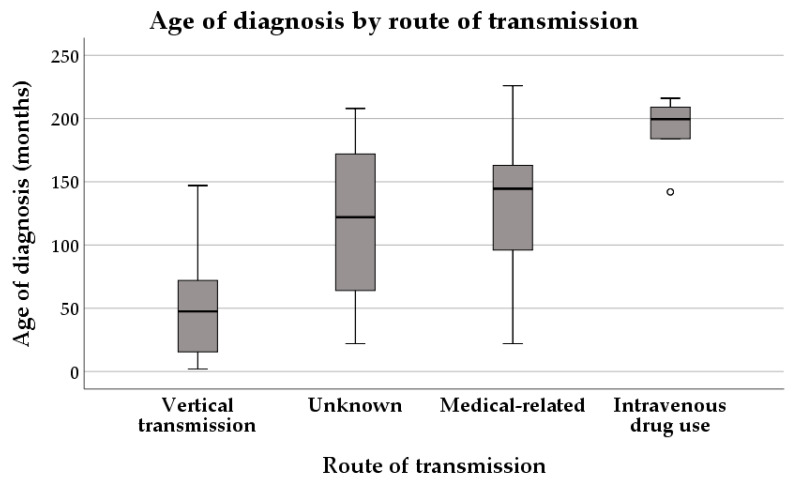
Age of diagnosis by route of transmission.

The patients with vertical transmission were younger than the others at the time of diagnosis, and the oldest patients were those with intravenous drug use as route of transmission (*p* < 0.001) (Table 3, Figure 7).

There was no statistically significant association detected between fibrosis severity and age at diagnosis (*p* = 0.475) or route of transmission (*p* = 0.269).

There was no statistically significant association detected between viral load and route of transmission (*p* = 0.386).

## 4. Discussion

The present retrospective study represents one of the few comprehensive analyses of pediatric hepatitis C in Eastern Europe, offering insight into the epidemiological and clinical features of 83 children with chronic HCV hepatitis over three decades [20,21,22]. Despite a decrease in HCV prevalence in some countries, with Thomadakis et al. (2019) estimating around 2.2% and Hleyhel et al. (2023) 0.9%, Romania still reports higher rates than most developed countries, and data regarding the pediatric population remain limited [13,14,23]. It is important to note that our hospital is the only center in the capital city of Romania and the surrounding areas providing access to treatment and follow-up for pediatric patients with HCV infection.

The predominance of patients in our cohort who were diagnosed between 2010 and 2019 is most likely due to improvements in awareness of pediatric HCV infection and the wider availability of targeted testing strategies, rather than representing a true increase in disease incidence. During this period, heightened recognition of vertical transmission and advances in diagnostic assays may have contributed to the more frequent identification of infected children. Consequently, this temporal clustering of diagnoses likely reflects changes in clinical practice and surveillance rather than the epidemiologic expansion of HCV infection. In most cases, hepatitis C is asymptomatic and slowly progresses over several decades, and because of this, many patients still remain undiagnosed [6,24]. Despite campaigns meant to increase awareness of HCV infection, there are studies that estimate a decreased awareness over the last years in the general population and even among physicians [25,26,27,28,29,30]. It is estimated by the WHO that only 20% of the patients are aware of the diagnosis [31].

Vertical transmission was the most identified route in our cohort (48.2%), and patients with perinatal acquisition were significantly younger at diagnosis compared to those with other transmission routes, such as intravenous drug use or iatrogenic/medical exposure. These findings emphasize the need for routine maternal HCV screening during pregnancy, in accordance with WHO recommendations, which remains lacking in Romania [32]. Being unaware of the disease increases the risk of transmission, and for the pediatric population it is very important to diagnose the disease in people of reproductive age [31].

We reported a slight female predominance, similar to the results of other studies [33].

CDC recommends using an HCV antibody test for all perinatally exposed children at the age of 18 months, while HCV-RNA can be used in children as early as 2 months of age. In our cohort the median age at diagnosis was 73 months, with most children being diagnosed beyond 2 years of age (83.1%), which reflects the lack of diagnosis in pregnant women and lack of further surveillance of children in our country. Another pediatric research conducted in Italy that included patients with HCV reports a median age at diagnosis of 8 years, but in patients vertically infected, most were diagnosed before 18 months (60%). However, failure to identify infected children from HCV-positive mothers has been previously reported [34,35,36].

It is well known from previous research that patients with hepatitis C have normal or minimally elevated transaminases, which is also the conclusion of this current research [37].

Fibrosis was not evaluated in all patients in our research because FibroScan^®^ was not largely accessible throughout the years, and the invasive nature of liver biopsy made it less widely used. As previously reported, our research supports the conclusion that most children with hepatitis C have only mild liver scarring. Other research report minimal or low-grade fibrosis in 95% of patients with hepatitis C acquired in childhood. However, in our cohort the percent of patients with moderate liver scarring was higher than reported by NASPGHAN (33.7% vs. 4%). The relatively high proportion of F2 fibrosis (33.7%) observed in our cohort should be interpreted with caution. Liver stiffness thresholds in children are not fully standardized, and transient elastography values may be influenced by age, inflammatory activity, and transaminase levels. Therefore, some degree of overestimation cannot be entirely excluded. Differences in applied cut-off values across studies may partially explain the discrepancy among our findings and previously published pediatric cohorts. In our study group there was one patient with cirrhosis at the time of diagnosis, which is rarely reported in pediatric hepatitis C. Although in most pediatric cases it is generally considered that HCV hepatitis advances slowly, there are cases with rapid progression towards cirrhosis and end-stage liver disease, as illustrated by this respective research [38,39,40]. This finding challenges the long-held perception that HCV progresses slowly and benignly in children and supports early identification and monitoring, especially in those infected perinatally [41,42,43].

Four (4.2%) patients included in our study group had HBV coinfection, which has been reported to increase severity of the disease. However, this research did not identify any association between HBV coinfection, level of transaminases or viremia, and fibrosis severity, these findings not suggesting more severe disease for coinfected patients at the time of diagnosis [6].

In children, the most commonly stated route of transmission is perinatal, and the epidemiology of hepatitis C greatly depends on seroprevalence during pregnancy [6]. This fact is supported by our research, which reports that patients with vertical transmission were significantly younger when compared to those with other routes of transmission. However, in our cohort, for one third of the patients (32.5%), the route of transmission remained unknown. This may be partially attributed to retrospective data limitations and underreporting, but it also highlights gaps in the diagnostic pathway and the need for improved risk factor assessment in pediatric settings.

The inadequate prenatal screening and the high number of unmonitored pregnancies in our country point to the lack of education, information, and financial means of the population. As such, children with chronic hepatitis C, who are mostly asymptomatic, are not properly assessed, leading to a late diagnosis, sometimes when fibrosis is already present. We can consider that, even though involuntary, this may be seen as a form parental neglect [44].

Given that we did not identify a significant association between viral load and transaminase levels in children with positive viremia, it appears that viral replication alone does not directly impact the degree of hepatocellular injury in children with HCV hepatitis. Additionally, the fact that there was no correlation identified between liver fibrosis severity and either age or route of transmission suggests that disease progression cannot be reliably predicted based on these factors, and individual factors might be essential. Early diagnosis and treatment are of utmost importance.

Together, these findings emphasize the fact that liver disease in pediatric HCV patients is complex and multifactorial, and its evolution is likely influenced by a combination of factors. Therefore, due to this high variability, it is not accurate to rely on single clinical or virological markers to assess disease severity in these patients. Complex, long-term follow-up and evaluation of these patients is needed.

HCV genotyping was not available for the patients in our cohort, as this investigation was not routinely supported by the national health system during most of the study period. In Romania, genotype 1b has historically been the predominant strain, accounting for the vast majority of infections, followed by genotypes 1a [45,46,47].

In Romania, treatment for chronic HCV hepatitis is currently provided through a national health insurance program, which ensures access to interferon-free direct-acting antiviral (DAA) regimens [13,48]. Initially, in our country, the combination ledipasvir/ sofosbuvir was exclusively used for children older than 12 years, but currently the pan-genotypic therapies—sofosbuvir/velpatasvir and glecaprevir/pibrentasvir—are available for children older than 3 years and 12 years, respectively, and achieve SVR rates of 100% [49]. However, access to treatment in children has historically been delayed compared to adults, and structured perinatal screening remains insufficient [22].

### 4.1. Limitations

The retrospective nature of this study inherently limits the accuracy of some variables, such as transmission route, and precludes long-term outcome assessment. Incomplete FibroScan^®^ data further limit interpretation.

The long study period represents an additional limitation. Over three decades, diagnostic assays, fibrosis assessment tools, and screening recommendations have evolved, potentially introducing temporal heterogeneity. Earlier cases may have been diagnosed using assays with higher detection thresholds, and transient elastography was not uniformly available throughout the entire study period. Although all data were analyzed at the time of diagnosis, these methodological variations may affect direct comparability across decades.

The absence of universally validated pediatric-specific transient elastography cut-offs may limit direct comparability with other cohorts.

HCV genotyping was not available, as it was not supported by the national health system, so most patients were unable to afford it and it was not mandatory for therapy.

Treatment outcome data, including SVR rates and access to DAA therapies, were not available for the majority of patients in this cohort, as most were diagnosed and followed up prior to or during the early transition to the DAA era in Romania.

### 4.2. Future Perspectives

Improving pediatric HCV outcomes in Eastern Europe will require the timely national adoption of international treatment guidelines, universal access to interferon-free DAA regimens, and the implementation of systematic perinatal screening programs. Future research should focus on evaluating real-world treatment outcomes with DAAs and assessing the impact of policy interventions on earlier diagnosis. Moreover, the development of a HCV vaccine remains a critical long-term goal for global eradication efforts [50,51,52]. Future prospective studies should incorporate genotyping data, particularly in the current era of pan-genotypic DAA [53,54].

## 5. Conclusions

This retrospective study provides a comprehensive overview of pediatric hepatitis C in the southern region of Romania over a period of more than three decades. The findings highlight the changing epidemiological landscape, with vertical transmission remaining the dominant route of infection, and a significant proportion of cases still diagnosed late, particularly among adolescents with risk factors, such as parenteral exposure or intravenous drug use. Although pediatric HCV infection is generally considered mild, only 40.6% of children had absent or mild fibrosis at diagnosis, while 33.7% already presented moderate fibrosis, and 8.4% had severe fibrosis or cirrhosis.

## Figures and Tables

**Table 1:** Age distribution at diagnosis.

Age at Diagnosis	Frequency (*n*)	Percentage (%)
**Under 2 years (*n* = 14)**		
0–6 months	2	14.3%
6–12 months	2	14.3%
12–18 months	7	50.0%
18–24 months	3	21.4%
**2 years and above (*n* = 69)**		
2 years	4	6.0%
3 years	7	10.4%
4 years	6	9.0%
5 years	6	9.0%
6 years	5	7.5%
7 years	1	1.5%
8 years	6	9.0%
9 years	5	7.5%
10 years	2	3.0%
11 years	2	3.0%
12 years	2	3.0%
13 years	6	9.0%
14 years	4	6.0%
15 years	3	4.5%
16 years	1	1.5%
17 years	7	10.4%
**Total**	**83**	**100%**

**Table 2:** Descriptive statistics: main characteristics of the population.

Variable	N (%)	Median (IQR)
**Decade of diagnosis**		
1990–1999	10 (12%)	
2000–2009	22 (26.6%)	
2010–2019	48 (57.8%)	
2020–2025	3 (3.6%)	
**Gender**		
Male	38 (45.8%)	
Female	45 (54.2%)	
Age		73 (36–156) months
**Area of provenance**		
Urban	62 (74.7%)	
Rural	21 (25.3%)	
**Route of transmission**		
Vertical	40 (48.2%)	
Medical-related	10 (12%)	
Intravenous drug use	6 (7.2%)	
Unknown	27 (32.5%)	
Alanine aminotransferase (ALT)		52 (27–84) IU/L
Aspartate transaminase (AST)		46.5 (26.25–78.25) IU/L
Viral load		3 × 10^5^ (1.1 × 10^5^–9 × 10^5^)
**Fibrosis**		
Unavailable	14 (16.9%)	
Available	69 (83.1%)	
F0–F1	34 (40.6%)	
F2	28 (33.7%)	
F3	6 (7.2%)	
F4	1 (1.2%)	
**HBV coinfection**		
Yes	4 (4.8%)	
No	79 (95.2%)	

**Table 3:** Median age at diagnosis by route of transmission.

Potential Route of Transmission	Median Age (Months)	Kruskal–Wallis Test for Independent Samples (*p*)
Vertical transmission	47.5 (15.25–72)	<0.001
Medical-related transmission	144.5 (87.75–169.5)	
Intravenous drug use	199.5 (173.5–210.75)	
Unknown route of transmission	122 (64–172)	

## Data Availability

The original contributions presented in this study are included in the article. Further inquiries can be directed to the corresponding author.

## Abbreviations

The following abbreviations are used in this manuscript:

HCV	hepatitis C virus
ALT	alanine aminotransferase
AST	aspartate transaminase
